# Does timely reporting of preoperative CT scans influence outcomes for patients following emergency laparotomy?

**DOI:** 10.1308/rcsann.2023.0040

**Published:** 2024-06-13

**Authors:** S Ikram, N Mirtorabi, D Ali, H Aain, DN Naumann, M Dilworth

**Affiliations:** University Hospitals Birmingham NHS Foundation Trust, UK

**Keywords:** Laparotomy, CT scan, NELA, Mortality

## Abstract

**Introduction:**

Timely preoperative computed tomography (CT) scans are important for patients requiring emergency laparotomy. United Kingdom guidelines state that a CT scan should be reported within 1h for ‘critical’ patients (will alter management at the time) and within 12h for ‘urgent’ patients (will alter management but not necessarily that day).

**Methods:**

An observational study included patients who were added to the National Emergency Laparotomy Audit (NELA) at a National Health Service trust from 2014 to 2021. The association of compliance with timings guidance and mortality was investigated. Multivariable logistic regression was used to determine the odds ratio of adherence to guidelines according to age, gender, night time admission, American Society of Anesthesiology (ASA) score, NELA mortality risk and category of scan. Further models determined the influence of adherence to guidelines on mortality, also adjusted for these variables.

**Results:**

There were 1,299 patients (48% ‘critical’ and 52% ‘urgent’ CT scans). Only 360/1,299 (28%) of scans were undertaken with adherence to the timing guidelines. Critical scans were less likely to adhere to guidelines. Although univariable analysis suggested that adherence to guidelines was associated with reduced mortality, this was not the case in the multivariable model: only age, ASA and NELA mortality risk remained significantly associated with mortality.

**Conclusions:**

A minority of patients met the recommended preoperative CT report timings, and this was less likely for scans designated ‘critical’. This did not appear to affect mortality when adjusted for key variables of risk. This illustrates the phenomenon of guideline adherence appearing to affect patient outcomes as a product of selection bias rather than causality.

## Introduction

Computed tomography (CT) has an essential role in diagnosing surgical pathology and devising appropriate management plans (both operative and nonoperative). Early CT scanning after admission to hospital is increasingly used before surgery for acute surgical abdominal pathologies. Rapid diagnosis followed by intervention is likely to have an impact on patient outcomes.^1^

In the United Kingdom (UK), the use of CT imaging in the preoperative period is suggested as being associated with decreased mortality for high-risk surgical patients, and is a minimum standard in the emergency laparotomy patient pathway.^2^ The best practice guidelines used to define the clinical standards for CT scan timings were published within the ‘NHS Services, Seven Days a Week Forum’ and the Royal College of Surgeons of England document ‘Emergency Surgery Guidance for Providers, Commissioners and Service Planners; Standards for Unscheduled Surgical Care’.^3,4^ These guidelines state that a CT scan should be reported within 1h of request for ‘critical’ patients (when the test will alter their management at the time) and within 12h for ‘urgent’ patients (if the test will alter their management but not necessarily that day).

It has been reported that there is a higher risk of mortality with delays in operative intervention and source control for gastrointestinal perforation.^5,6^ Some of the delay in emergency surgery may be attributed to delays in preoperative diagnostic imaging.^7^ A study from the USA reported that delays in preoperative CT scanning can have adverse outcomes in the elderly population and higher complication rates.^8^ Although early preoperative imaging is an audit standard according to the National Emergency Laparotomy Audit (NELA), a study of the association between the time taken for imaging and patient outcomes for those undergoing emergency laparotomy has not yet been undertaken in the UK.

The aim of the current study was to investigate the relationship between timing of CT (from request to report) and outcomes for patients who require emergency laparotomy, to determine whether this standard is justified for this patient group. We hypothesised that patients with delayed CT imaging may have worse outcomes than those who underwent CT scanning within the audit standards.

## Methods

### Study design and setting

An observational study was undertaken to examine the relationship between adherence to best practice guidance on CT scan timings (from request to report) and patient mortality. This was done using a database extracted from a single site’s NELA records between January 2014 and December 2021. There were no changes in the number or availability of CT scanners or emergency operating theatres during the study period. Institutional approval was granted before data collection.

### CT timing guidance

For the current study, the category of scan was taken directly from the NELA records (the study investigators did not make their own judgements about the scan categories). These were categorised into ‘critical’ or ‘urgent’. Scans were considered to meet the guideline if they were reported for these categories within 1 and 12h of the scan request, respectively. Because the category of scans was taken from the NELA records, this was not declared at the time of the CT request, and the categorisation is not available on the CT requesting system at the NHS trust. Urgency of scanning at this trust is usually relayed to the radiologist on-call via electronic request and telephone call.

### Data collected

All data were taken directly from the NELA records for patients. These included demographic details (age, gender), physiological status, American Society of Anesthesiology (ASA) score and NELA mortality risk prediction, malignancy status, timing and category of scans (critical or urgent), timing of admission (day or night, defined as 8am to 8pm and 8pm to 8am, respectively), and adherence to timings guidance. Timings were calculated by measuring the period between the CT scan being requested and reported. The primary outcome of interest was 30-day mortality, which was taken from the NELA records.

### Statistical analysis

Continuous data are summarised as median and interquartile range (IQR), and categorical data are summarised as number (%). Simple pairwise univariable analyses were undertaken using the Mann–Whitney *U* test for continuous variables and chi-squared analysis for categorical variables. Separate univariable and multivariable logistic regression models were used to determine the odds ratio (OR) and 95% confidence intervals (95% CI) for both adherence to the CT timings standard and 30-day mortality. All models were undertaken using independent variables that were selected a priori because of their credible influence on the dependent variables of interest, and included age, sex, nighttime admission, NELA mortality prediction and ‘critical’ status. A value of *p* < 0.05 was considered statistically significant. Analyses were undertaken using GraphPad Prism (version 9.4; GraphPad Software) and R (version 1.4; R Foundation for Statistical Computing, Vienna, Austria).

## Results

### Study patient characteristics

There were 1,299 patients with a median age of 66 (IQR 52–76) years; 626/1,299 (48%) were male. Patient characteristics are summarised in Table 1 and compared between those who died within or survived for 30 days. On pairwise univariable analysis, patients who died within 30 days were more likely to be older, have a higher ASA score, greater NELA mortality risk, and were more likely to require a critical scan than those who survived (Table 1). Those who survived were more likely to have had a CT scan that adhered to the timings standard than those who died.

**Table 1:** Patient characteristics for survivors and those who died within 30 days

Characteristic	All	Survived	Died	*p*-value
	(*N* = 1,299)	(*n* = 1,157)	(*n* = 142)	
Age	66 (52–76)	65 (50–76)	72 (64–80)	**<0.001**
Male gender	626 (48)	557 (48)	69 (49)	0.919
Admitted at night	458 (35)	409 (35)	49 (35)	0.843
ASA	3 (2–3)	3 (2–3)	3 (3–4)	**<0.001**
NELA risk %	6 (3–19)	4 (2–12)	29 (14–63)	**<0.001**
Malignancy	273 (21)	241 (21)	32 (23)	0.638
CT scan category				**<0.001**
Critical	622 (48)	526 (45)	96 (68)	
Urgent	677 (52)	631 (55)	46 (32)	
Adhered to CT guidance	360 (28)	333 (29)	27 (19)	**0.014**

ASA = American Society of Anesthesiologists; CT = computed tomography; NELA = National Emergency Laparotomy Audit

Continuous data are given as the median with interquartile range in parentheses; categorical data are given as *n* (%)

### CT timings

**Figure 1:**
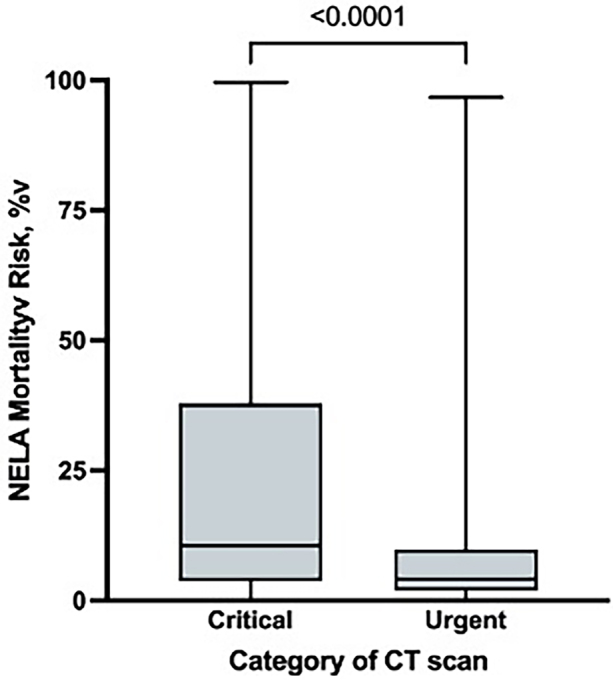
Distribution of National Emergency Laparotomy Audit mortality risk according to category of computed tomography scan

**Figure 2:**
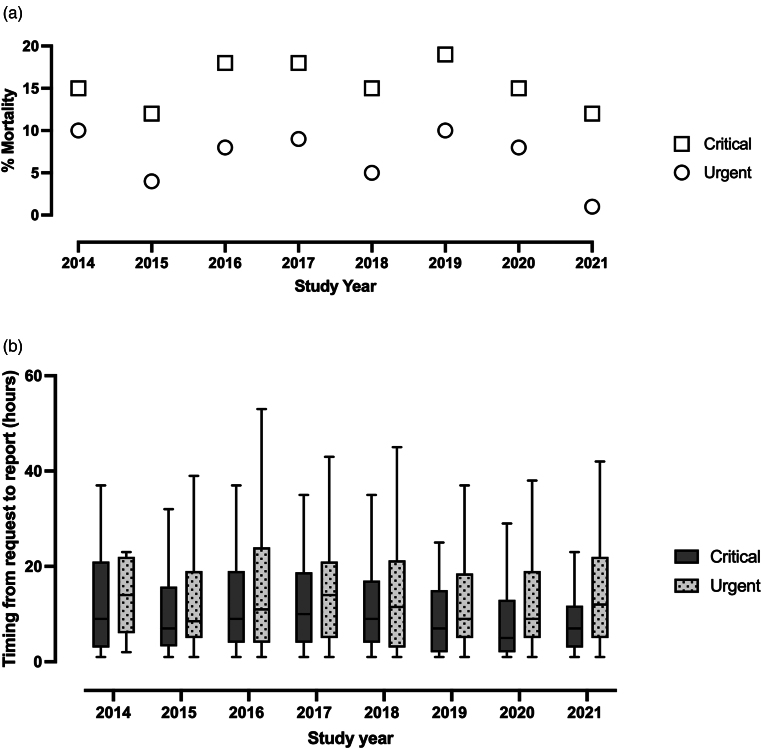
Summary of the (a) mortality rate and (b) timings from computed tomography request to report throughout the study period for both ‘critical’ and ‘urgent’ scan subgroups. Box and whisker plots are displayed using the Tukey method with the horizontal line representing the median, the box indicating the interquartile range (IQR) and the whiskers as 1.5 IQR.

There were 622/1,299 (48%) critical and 677/1,299 (52%) urgent CT scans during the study period. A CT scan that was classified as critical was associated with higher NELA mortality risk compared with a CT scan that was classified urgent (11% [IQR 4–38%] vs 4% [IQR 2–10%] respectively; *p* < 0.001) (Figure 1). Only 360/1,299 (28%) of scans were undertaken within the timings standard, including 359 of 677 (53%) urgent and 1 of 622 (0.2%) critical scans (*p* < 0.001). The median time between request and report was 8h 15min (IQR 3h 48min to 16h 38min) for critical scans and 11h 2min (IQR 5h 12min to 20h 48min) for urgent scans. There was no significant trend in timings over the study period (Figure 2). On univariable logistic regression analysis, patients were less likely to have a scan within the timings standard if they had higher ASA scores, greater NELA risk of mortality and a scan that was classified as critical (Table 2). When a multivariable logistic regression model incorporated age, gender, nighttime admission, ASA, NELA mortality risk and category of scan, patients remained less likely to have a scan within the timings standard if it was classified as critical (Table 2). They also appeared to be more likely to adhere to guidance during the night (Table 2).

**Table 2:** Odds ratio of adherence to computed tomography scan timing standard according to patient characteristics and timing of admission using univariable and multivariable logistic regression models

	Univariable analysis			Multivariable analysis		
Characteristic	OR	95% CI	*p*-value	OR	95% CI	*p*-value
Age	1	0.99, 1.01	0.623	1	0.99, 1.01	0.409
Male gender	0.99	0.78, 1.27	0.952	1.17	0.86, 1.60	0.305
Admitted at night	1.24	0.197, 1.60	0.09	1.87	1.35, 2.61	**<0.001**
ASA	0.78	0.78, 0.89	**<0.001**	1.05	0.85, 1.30	0.643
NELA risk %	0.97	0.96, 0.98	**<0.001**	1	0.98, 1.01	0.739
Critical scan	<0.01	<0.01, <0.01	**<0.001**	<0.01	<0.01, <0.01	**<0.001**

ASA = American Society of Anesthesiologists; CI = confidence intervals; CT = computed tomography; NELA = National Emergency Laparotomy Audit; OD = odds ratio

### Thirty-day mortality

Some 142/1,299 (11%) patients died within 30 days. There was no significant trend in mortality over time (Figure 2). Mortality at 30 days was associated with poorer adherence to CT timings standard (Table 1). However, for the 677 patients in the subgroup who had urgent scans, 30-day mortality was 27/359 (8%) for those who met the standard and 19/318 (6%) for those who did not (*p* = 0.425). On univariable logistic regression analysis, patients were more likely to die within 30 days if they were older, had a higher ASA score and greater NELA risk of mortality (Table 3). When a multivariable logistic regression model was used to incorporate age, gender, nighttime admission, ASA, NELA mortality risk, malignant status and adherence to timings standard, adherence to timings was no longer a statistically significant independent variable; instead, only age, ASA and NELA mortality risk remained statistically significant (Table 3).

**Table 3:** Odds ratio of 30-day mortality according to adherence to computed tomography scan timing standard using univariable and multivariable logistic regression models that include age, gender, admission at night, ASA, NELA risk and malignancy status

	Univariable analysis			Multivariable analysis		
Characteristics	OR	95% CI	*p-*value	OR	95% CI	*p-*value
Adherence to CT timing guidance	0.58	0.37, 0.89	**0.015**	0.89	0.54, 1.43	0.652
Age	1.04	1.02, 1.04	**<0.001**	1.03	1.01, 1.04	<**0.001**
Male gender	1.02	0.72, 1.44	0.919	0.9	0.61, 1.33	0.606
Admitted at night	0.96	0.66, 1.38	0.843	0.84	0.56, 1.24	0.385
ASA	2.53	2.04, 3.17	**<0.001**	1.68	1.32, 2.15	**<0.001**
NELA risk %	1.04	1.03, 1.04	**<0.001**	1.03	1.02, 1.04	**<0.001**
Malignancy	1.11	0.72, 1.66	0.638	0.99	0.62, 1.54	0.953

ASA = American Society of Anesthesiologists; CI = confidence intervals; CT = computed tomography; NELA = National Emergency Laparotomy Audit; OD = odds ratio

## Discussion

The main finding from the current study is that adherence to the best practice standard for the timing of a CT scan from request to report appears at first to be associated with better 30-day survival, but this finding is no longer significant in the adjusted models. This appears to be a result of scans being less likely to adhere to guidance for patients who were at higher risk (higher NELA mortality risk, higher ASA scores and more likely to be categorised as ‘critical’). Sicker patients appeared to be less likely to have a CT scan that adhered to guidance, and also more likely to die within 30 days. Even though the data demonstrate that patients receiving CT scans more quickly were less likely to die within 30 days, it appears that it is not the only affecting factor.

CT scans form the mainstay mode of investigating patients presenting with an acute abdomen and can support decision making in patients who might require laparotomy. The fourth NELA report was the first to recommend that patients who require immediate surgical management should not be delayed by waiting for a CT scan.^9^ Other authors have reported improved outcomes with early CT scans for patients undergoing emergency laparotomy for trauma.^10^ There is some evidence that earlier CT scans for better diagnostic accuracy and decision making reduce time for operative intervention.^10,11^ Gil-Sun Hong *et al* report improvement in intensive care unit admissions with a dedicated radiology team for emergency surgery, which was associated with faster scans and earlier operative management.^12^ However, the current study demonstrates that the relationship between timings and outcomes for emergency non-trauma laparotomy is more complex and is influenced by other patient factors.

In the trauma setting, there is similar evidence that the timing of CT scans is not as significant a factor as other patient factors such as age and injury severity, and that patients who undergo scanning within the guidelines are not necessarily matched with those who do not.^13^ Some investigators have looked at the physical distance of the CT scanner from the trauma room and found that reduced CT scan timing and improved survival outcomes were noted in groups in which the CT scan was located within 50m.^14^ Other studies report improved outcomes and time benefits in patients with both penetrating and blunt injuries who had prompt preoperative CT scans^10,15,16^ enabling timely management.

The current study suggests patients who are more unwell are more likely to miss the 60min window standard for reporting a ‘critical’ CT scan that has been recommended by NELA. Indeed, the fourth NELA report advocates a 60min window for all scans for laparotomy patients (regardless of category).^9^ If this were applied then there may be even poorer compliance, with an unknown effect on patient outcomes. The current study illustrates one problem with imposing audit standards on practice with unknown or unproven influence on outcomes. Further investigations are required to determine which of the individual NELA audit standards are likely to improve patient outcomes and which might just reflect the overall clinical picture and be less helpful.

An important disadvantage of the NELA categorisation of ‘urgent’ and ‘critical’ scans is that these categories are not universally declared within the CT requesting system or in the logistics of moving patients to and from the CT scanner. Instead, they are input into the online audit form, usually after the event. Therefore, it may not always be clear which timing standard applies at the time of the request. From a practical standpoint, surgical patients requiring early operative intervention will continue to undergo CT imaging as early as possible to increase preoperative diagnostic accuracy. Although this study demonstrates that most scans were not reported within the standard time, this did not seem to impact this pathway or the outcome. These high-risk patients will have increased perioperative mortality risks caused by the disease process itself and associated patient factors.

### Study limitations

This study is observational and retrospective, with all the usual limitations of this design, such as selection bias, missing data and transcription errors. We were unable to determine whether there were any significant changes to the number or availability of radiologists or the availability of remote access during the study period. All clinical data for the study (such as ‘urgent’ vs ‘critical’) were taken verbatim from clinical records without any interpretation by the authors, and it is not known whether these categories were applied consistently between individuals. We were unable to determine the exact reason for laparotomy. Because of the retrospective design, we were not able to determine whether scans were performed within the timing standard but not yet reported. It is possible that such scans may have been seen by the surgical team and acted upon prior to a formal radiology report. We were also unable to determine whether the scan reports were accurate or whether they changed the management of the patients within the study cohort. Further investigations may determine whether there is a relationship between timings, accuracy of diagnosis and patient outcomes.

## Conclusion

In our study of 1,299 patients who required CT imaging before emergency laparotomy, guidelines for reporting were adhered to for only a minority of patients. However, there was no clear association between adherence to preoperative CT reporting guidelines and 30-day mortality. This may be because patients who were sicker were less likely to meet the timing standards, and also less likely to survive. This illustrates a selection bias when assessing patient outcomes according to guideline adherence in emergency surgery; adherence to audit standards may initially appear to be associated with improved outcomes but it is important to match patients for their disease and demographic characteristics when assessing these standards.

## References

[1] Giangola M, Havens JM. Imaging in emergency general surgery. In: Brown C, Inaba K, Martin M, Salim A. *Emergency General Surgery*. Cham: Springer; 2019. pp. 27–39.

[2] *The High-Risk General Surgical Patient: Raising the Standard*. RCS England; 2018.

[3] NHS Services. Seven Days a Week Forum Summary of Initial Findings. December 2013.

[4] Emergency Surgery. Standards for Unscheduled Surgical Care; Guidance for Providers, Commissioners and Service Planners. February 2011.

[5] Buck DL, Vester-Andersen M, Møller MH. Surgical delay is a critical determinant of survival in perforated peptic ulcer. *Br J Surg* 2013; **100**: 1045–1049. 23754645 10.1002/bjs.9175

[6] Ong M, Guang TY, Yang TK. Impact of surgical delay on outcomes in elderly patients undergoing emergency surgery: A single center experience. *World J Gastrointest Surg* 2015; **7**: 208–213. 26425270 10.4240/wjgs.v7.i9.208 PMC4582239

[7] North JB, Blackford FJ, Wall D *et al.* Analysis of the causes and effects of delay before diagnosis using surgical mortality data. *BJS* 2013; **100**: 419–425. 23225342 10.1002/bjs.8986

[8] Ricci KB, Oslock WM *et al.* Importance of radiologists in optimizing outcomes for older Americans with acute abdomen. *J Surg Res* 2021; **261**: 361–368. 33493888 10.1016/j.jss.2020.12.022

[9] Fourth Patient Report of the National Emergency Laparotomy Audit (NELA), December 2016 to November 2017.

[10] van den Hout WJ, Van Der Wilden GM, Boot F *et al.* Early CT scanning in the emergency department in patients with penetrating injuries: does it affect outcome? *Eur J Trauma Emerg Surg* 2018; **44**: 607–614. 10.1007/s00068-017-0831-5 PMC6096612 28868591

[11] Rosen MP, Sands DZ, Longmaid III HE *et al.* Impact of abdominal CT on the management of patients presenting to the emergency department with acute abdominal pain. *Am J Roentgenol* 2000; **174**: 1391–1396. 10789801 10.2214/ajr.174.5.1741391

[12] Hong G-S, Lee CW, Lee JH *et al.* Clinical impact of a quality improvement program including dedicated emergency radiology personnel on emergency surgical management: A propensity score-matching study. *Korean J Radiol* 2022; **23**: 878–888. 35926842 10.3348/kjr.2022.0278 PMC9434742

[13] Ng CLH, Kim J, Dobson B *et al.* Time to computed tomography: does this affect trauma patient outcomes? A retrospective analysis at an Australian major trauma centre. *ANZ J Surg* 2019; **89**: 1475–1479. 31689726 10.1111/ans.15470

[14] Huber-Wagner S, Mand C, Ruchholtz S *et al.* Effect of the localisation of the CT scanner during trauma resuscitation on survival – a retrospective, multicentre study. *Injury* 2014; **45**: S76–S82. 10.1016/j.injury.2014.08.022 25284240

[15] Katayama Y, Kitamura T, Hirose T *et al.* Delay of computed tomography is associated with poor outcome in patients with blunt traumatic aortic injury: A nationwide observational study in Japan. *Medicine (Baltimore)* 2018; **97**: e12112. 30170440 10.1097/MD.0000000000012112 PMC6392548

[16] Murao, S, Yamakawa K, Kabata D *et al.* Effect of earlier door-to-CT and door-to-bleeding control in severe blunt trauma: A retrospective cohort study. *J Clin Med* 2021; **10**: 1522. 33917338 10.3390/jcm10071522 PMC8038745

